# Efficacy and Tolerability of Extended-Duration Tonic Motor Activation for Treatment of Restless Legs Syndrome with Awakenings During Sleep

**DOI:** 10.3390/jcm15082845

**Published:** 2026-04-09

**Authors:** Hussein Alawieh, Kurtis J. Swartz, Stephanie K. Rigot, Jonathan D. Charlesworth

**Affiliations:** Noctrix Health Inc., Pleasanton, CA 94566, USA; kurtis.swartz@noctrixhealth.com (K.J.S.); srigot@noctrixhealth.com (S.K.R.)

**Keywords:** sleep disorder, neurological disorder, neuromodulation, peripheral nerve stimulation, bioelectronic, restless legs syndrome

## Abstract

**Background:** Restless legs syndrome (RLS) is a prevalent neurological sleep disorder that often impairs sleep maintenance. This single-arm, open-label study evaluated the efficacy, safety, and tolerability of extended-duration tonic motor activation (XD-TOMAC) in adults with RLS who experience frequent awakenings with symptoms. **Methods**: The study comprised three stages: Stage 1 (2 weeks of no intervention), Stage 2 (8 weeks XD-TOMAC), and Stage 3 (2 weeks of no intervention). XD-TOMAC consisted of bilateral high-frequency peroneal nerve stimulation programmed to 180 min duration and administered nightly at bedtime. Nineteen adults with moderate–severe RLS were enrolled, each reporting at least three nights per week of RLS symptoms causing increased awakenings or interfering with returning to sleep after waking. **Results**: The intent-to-treat analysis population included all patients who began Stage 2 (*n* = 15). After 8 weeks of XD-TOMAC, the mean change in International RLS Study Group Rating Scale (IRLS) score was −10.6 points (*p* < 0.001), and the mean change in Medical Outcomes Study Sleep Problems Index II (MOS-II) was −29.5 points (*p* < 0.001). The mean change in the number of nocturnal awakenings was −1.1 per night (*p* = 0.009), and the mean change in sleep efficiency was +8.5% (*p* = 0.001). The mean change in time awake with RLS symptoms after sleep onset was −28.1 min (*p* = 0.009). Each of these improvements was sustained at the end of Stage 3 (*p* < 0.01). There were no serious or severe device-related adverse events. **Conclusions**: Compared with prior 30 min TOMAC studies, XD-TOMAC demonstrated greater efficacy and similar tolerability, supporting its potential as a nonpharmacological therapy for RLS patients whose symptoms frequently disrupt sleep.

## 1. Introduction

Restless legs syndrome (RLS) is a common neurological sleep disorder characterized by an urge to move the legs that worsens during rest and in the evening or nighttime hours [1]. Sleep disturbance is a common morbidity of RLS, affecting both sleep initiation and maintenance [2,3,4]. Patients frequently experience prolonged sleep latency, recurrent nocturnal awakenings, and reduced sleep continuity, leading to chronic sleep loss and impaired daytime functioning [3,4,5,6]. Improving sleep is, therefore, a central therapeutic goal in RLS management.

Pharmacologic therapy has historically been the primary treatment for RLS; however, long-term effectiveness is limited for a substantial proportion of patients [7]. Dopamine agonists, once considered first-line therapy, are now conditionally recommended against in recent American Academy of Sleep Medicine (AASM) guidelines due to the risk of augmentation and loss of efficacy over time [8]. Alternative first-line agents, including α2δ ligands, are not associated with augmentation but may be limited by adverse effects and tolerability [8,9]. Off-label opioids remain an option for severe refractory RLS but are prescribed sparingly by most healthcare providers and have limited availability, partially due to CDC guidelines recommending against off-label opioid prescription [10,11]. Consequently, nonpharmacological alternatives would be beneficial.

To address this unmet need, tonic motor activation (TOMAC) was developed. TOMAC is a nonpharmacological therapy that delivers bilateral high-frequency stimulation to the peroneal nerve to evoke sustained increases in tibialis anterior muscle tone without producing overt limb movements, allowing therapy to be delivered during sleep [12,13,14]. TOMAC is conditionally recommended in the most recent AASM clinical practice guidelines for the treatment of RLS [8].

**Figure 1:**
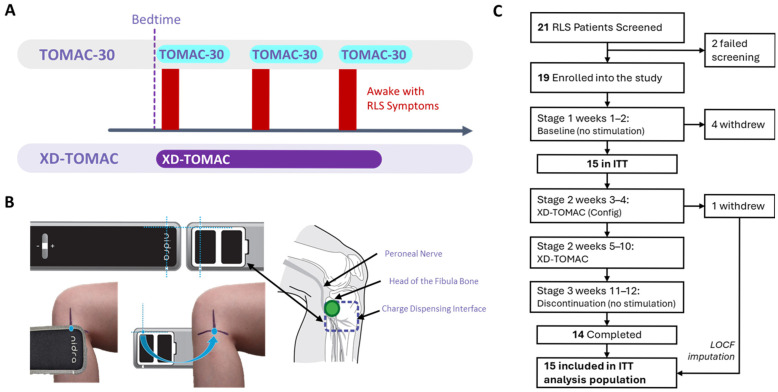
Participant disposition and study design. (**A**) Prior studies investigated TOMAC programmed to a 30-min duration (TOMAC-30) and activated as needed when the patient was awake with RLS symptoms. XD-TOMAC was programmed to a 180-min duration and administered nightly at bedtime. (**B**) The TOMAC system consists of bilateral therapy units for the right and left legs. Each unit delivered electrical stimulation via a charge-dispensing interface positioned over the peroneal nerve near the fibular head. (**C**) The study timeline comprised four stages: Stage 1 (no TOMAC), Stage 2 (XD-TOMAC), and Stage 3 (no TOMAC). XD-TOMAC = extended-duration tonic motor activation; LOCF = last observation carried forward; ITT = intention to treat; RLS = restless legs syndrome.

The efficacy and safety of TOMAC were demonstrated in the RESTFUL trial, a multicenter, randomized, double-blind, sham-controlled study in which participants self-administered 30 min TOMAC sessions as needed when RLS symptoms occurred, an approach we refer to henceforth as “TOMAC-30” [14,15]. While effective, one limitation of TOMAC-30 is that patients must wait until after they wake up to activate TOMAC-30. Recent analysis indicates that patients wait an average of 8.6 min after waking before deciding to activate TOMAC-30, and TOMAC-30 takes an average of 12.1 min to restore sleep [16]. Here, we sought to prevent these midnight awakenings as opposed to treating them after they occurred (Figure 1A).

In this study, we tested the hypothesis that administering TOMAC starting earlier in the night (before awakenings) for a much longer duration of 180 min (extended duration, XD-) would have a prophylactic effect, suppressing RLS-related awakenings and thereby improving RLS and sleep quality.

## 2. Materials and Methods

The study was conducted in accordance with the International Conference on Harmonization guidelines on good clinical practice and the Declaration of Helsinki. The protocol and informed consent were approved by a central institutional review board (Advarra, Columbia, MD, USA), and all participants provided informed consent. The first participant was enrolled on 11 March 2025, and the trial was registered with ClinicalTrials.gov (NCT06866132) on 6 March 2025.

### 2.1. Participants

Participants were recruited through online advertisements with materials approved by the institutional review board. Patients who responded to the online advertisements were then prescreened, screened, and enrolled in order. Key eligibility criteria were age (adults 22–79 years old), diagnosis of RLS confirmed based on the International RLS Study Group criteria [17], moderate–severe RLS defined by the International RLS Study Group Rating Scale (IRLS) total score ≥ 15, RLS symptoms being most significant in the lower legs and/or feet, and participant-reported RLS-related awakenings during sleep, which either caused the awakening or interfered with returning to sleep after being awake, in at least 3 nights per week during the month before study entry. Key exclusion criteria were unstable dosing of RLS medications, inadequately treated primary sleep disorders other than RLS, severe peripheral neuropathy involving the lower legs, dermatologic conditions at the stimulation application site, known hypersensitivity to device materials, presence of active implanted medical devices, epilepsy, highly irregular sleep schedules (defined as habitual bedtime outside the window of 9:00 PM to 3:00 AM or bedtime variability exceeding four hours), improper device fit or inability to achieve calibrated stimulation intensity within operational limits during initial device setup, and prior exposure to the study device or any neurostimulation therapy for RLS.

### 2.2. Study Design

This was a single-arm, open-label feasibility study conducted at a single site in the United States. An independent medical monitor, blinded to study stage and treatment parameters, adjudicated all adverse events (AEs). Outcome measures were assessed at study entry and at the completion of each study stage. The total study duration was 12 weeks and comprised three sequential stages. Participants were sent a self-administered daily electronic questionnaire to monitor adherence to study procedures at each stage and to capture self-reported sleep diary entries. The schedule of assessments (“follow-ups”) was the following: Day 14 (XD-TOMAC initiation, training, and baseline assessments), Day 17 (adverse events and compliance check), Day 28 (device optimization), Day 31 (adverse events and compliance), Day 42 (4-week outcomes), Day 56 (adverse events and compliance), and Day 70 (8-week outcomes and transition to no-stimulation). This frequency of scheduled assessments was comparable to the comparator study [14].

Stage 1 involved 2 weeks with no intervention to collect baseline daily sleep diaries for evaluating sleep, awakenings during sleep, and awakenings related to RLS.

Stage 2 involved 8 weeks of XD-TOMAC intervention, where the first 2 weeks comprised a run-in period to select XD-TOMAC frequency and the final 6 weeks comprised XD-TOMAC treatment with the selected settings. Throughout Stage 2, participants were instructed to activate XD-TOMAC once every night, with the option to also activate TOMAC-30 later in the night if symptoms recurred. All participants were instructed to start XD-TOMAC at bedtime unless they reported on their Stage 1 sleep diaries that greater than 20% of RLS-related awakenings occurred more than 3 h after bedtime, in which case a delayed timer was used to start XD-TOMAC. This delayed timing only applied to one participant, for whom XD-TOMAC initiation was delayed by 1.45 h after bedtime to align therapy delivery with their individualized peak window of RLS-related awakenings.

During the Stage 2 run-in period to select XD-TOMAC settings, all participants received XD-TOMAC with a 2000 Hz waveform due to electrical characterization that shows favorable power consumption and thus longer runtime on the rechargeable device battery. After the run-in period, participants who did not report much improvement or very much improvement on the Patient Global Impression of Improvement (PGI-I) (i.e., PGI-I score ≥ 3) were switched to the 4000 Hz waveform used in prior studies of TOMAC-30, while the others continued with the 2000 Hz waveform. The PGI-I is a 7-point patient-reported scale assessing overall perceived change in condition (i.e., RLS) since treatment initiation.

After the Stage 2 run-in period, participants continued XD-TOMAC for six more weeks. Following completion of the nightly XD-TOMAC session, the investigational TOMAC device automatically reverted to standard 30 min session duration, permitting additional on-demand therapy sessions if RLS symptoms recurred later in the night. These additional sessions were manually started if the participant was awake and experiencing RLS symptoms after the XD-TOMAC session had ended. For the one participant with delayed XD-TOMAC initiation, an additional 30 min session was permitted at bedtime if RLS symptoms were present prior to sleep onset. All participants were instructed to activate XD-TOMAC daily, independent of symptom severity or timing. Primary and key secondary outcomes were assessed at the end of Stage 2 compared to study entry.

Stage 3 involved 2 weeks with no treatment to evaluate if any benefits of XD-TOMAC persisted after discontinuation. Throughout all study stages, participants completed daily questionnaires capturing RLS symptom severity and frequency and sleep parameters.

### 2.3. Investigational Device

The TOMAC system is positioned over the head of the fibula (Figure 1B) and delivers electrical stimulation to afferent fibers of the common peroneal nerve, producing sustained increases in tibialis anterior muscle tone without eliciting overt limb movements that could disrupt sleep [12]. This neuromodulatory approach engages pathways associated with voluntary leg activation to provide sleep-compatible reduction in RLS symptoms. Standard TOMAC therapy consists of patient-initiated 30 min stimulation sessions (TOMAC-30), which can be administered at symptom onset during waking hours, at bedtime, or following sleep onset. The system comprised two configurable, battery-powered, wearable neurostimulation units, one for each leg. Each unit delivered a proprietary electrical stimulation waveform through disposable adhesive hydrogel electrode patches placed superficially below the knee over the peroneal nerve near the head of the fibula.

Prior to at-home use, participants received standardized instruction on device placement and operation and completed either an in-person or remote video-based titration session. During titration, stimulation intensity was programmed to the maximum level that was comfortable and non-distracting within an allowable range of 0–40 mA. Participants were instructed to use the titrated intensity during therapy but were permitted to adjust stimulation within an approximate ±10% to 20% range to maintain comfort or relief, if needed.

For XD-TOMAC, stimulation duration was set to 180 min, corresponding to the maximum duration supported by a single battery charge using the power-efficient waveform at typical titrated comfort levels. Participants were instructed to initiate XD-TOMAC nightly at bedtime, irrespective of symptom presence. Following completion of the extended-duration session, devices automatically reverted to standard 30 min session mode, allowing participants to administer additional TOMAC-30 sessions as needed and if battery permitted for nocturnal RLS symptoms. For the participant assigned to delayed XD-TOMAC, therapy initiation was controlled using a Bluetooth-enabled timer application on a study-provided laptop, which automatically triggered stimulation at a predefined delayed interval after bedtime based on individualized symptom timing.

### 2.4. Efficacy Outcome Measures

All primary and key secondary efficacy outcome measures were prespecified patient-reported questionnaires assessed at the end of Stage 2 (after 8 weeks of XD-TOMAC) compared to study entry. The primary efficacy endpoint was the change in mean IRLS score. The key secondary efficacy endpoints were mean change in frequency of RLS symptoms (days per week), mean change in Medical Outcomes Study Sleep Problems Index II (MOS-II), mean change in MOS Sleep Problems Index I (MOS-I), and Participant Global Impressions of Improvement (PGI-I) responder rate. The IRLS and PGI-I were assessed over the previous week, and the MOS was assessed over the previous 2 weeks. The number of days with RLS symptoms per week (at any time of day) was derived from a follow-up to question 7 of the IRLS questionnaire. The IRLS is a 10-item questionnaire with scores ranging from 0 (no RLS symptoms) to 40 (most severe possible RLS symptoms). The MOS-I (6-items) and MOS-II (9-items) are the subscales of the 12-item MOS Sleep Scale, with scores ranging from 0 (no sleep problems) to 100 (worst possible sleep problems). PGI-I responder rate is defined as the proportion of participants with a response of “very much improved” or “much improved”.

### 2.5. Sleep Diary Outcome Measures

Sleep diary outcome measures were based on patient responses to daily electronic questionnaires during Stage 2 (XD-TOMAC) and Stage 3 (no intervention) compared to Stage 1 (no intervention). Participants reported their bedtime, morning wake-up time, sleep onset latency (SOL), total number of midnight awakenings during sleep time, number of awakenings with RLS during sleep time, time awake after sleep onset (WASO), and time awake with RLS after sleep onset. Time in bed (TIB) was computed as the duration between bedtime and wake-up time. Total sleep time (TST) was computed as (TIB-SOL-WASO), and sleep efficiency percentage (SE) was calculated as 100% × (TST/TIB).

### 2.6. Statistical Analysis

Efficacy analyses were conducted in the intention-to-treat (ITT) population. Hierarchical testing was used to control for multiplicity across prespecified endpoints, and two-sided tests were employed with an alpha level of 0.05. Changes in IRLS, MOS-II, and MOS-I were analyzed with one-sample *t*-tests, and PGI-I responder rates were compared using a two-sided normal approximation test. For participants who exited in Stage 2, data from earlier time points in Stage 2 (week 2 or 4) were imputed using last observation carried forward; if no such data were available, multiple imputation was applied. Since this was a feasibility study, the sample size was not strictly determined based on power analysis. However, it was noted prior to study start that a sample size of 10 participants would provide 90% power to detect significant IRLS improvement if the mean and variance were similar to the RESTFUL study [14]; sample size was increased above this minimum to improve precision. Continuous outcomes were analyzed using analysis of covariance (ANCOVA) to compare results across study conditions and evaluation time points, with baseline values included as covariates. Post hoc analyses were performed using two-sided *t*-tests with an alpha level of 0.05. When assumptions of normality were not met, appropriate nonparametric alternatives were applied. The Kolmogorov–Smirnov test was used to compare distributions across study conditions. All analyses were performed using Python 3.11.9.

### 2.7. Safety Analysis

Safety was evaluated by assessing the frequency and severity of device-related adverse events. Adverse events were classified as device-related if they had a temporal relationship to device usage and/or were an anticipated outcome associated with noninvasive electrical stimulation. The safety analysis population included all participants in the ITT population.

### 2.8. Exploratory Analysis

Exploratory analyses were conducted to determine which components of sleep were improved by XD-TOMAC at the end of Stage 2. Post hoc *t*-tests were conducted for each MOS item. For each item, a two-sided *t*-test with an alpha level of 0.05 was conducted, and the Holm–Bonferroni method was used to account for multiple comparisons. For each outcome metric based on subjective reporting from the daily sleep diary, values were first averaged within participant and then aggregated across participants. The sleep diary questionnaire items are described in Supplementary Material Section S1.

Since this study did not include a comparator group receiving TOMAC-30 therapy, a matched subset of participants who received TOMAC-30 for 8 weeks in RESTFUL. This matched subset compared outcomes from XD-TOMAC in the present study to outcomes from TOMAC-30 in the RESTFUL study. The comparator cohort comprised RESTFUL study participants assigned to 8 weeks of active TOMAC therapy who reported 2–3 nights of RLS symptoms per week in response to a baseline questionnaire item assessing the frequency of restless legs syndrome (RLS) symptoms after falling asleep (“Frequency [days/week]: 0–1, 2–3, 4–5, 6–7”). We compared distributions of the following outcomes that were collected at week 4 and week 8 in both studies: IRLS score, MOS-II score, and PGI-I responder rates.

## 3. Results

### 3.1. Participant Characteristics

Between 6 March 2025 and 25 April 2025, 21 individuals were screened for eligibility; two were deemed ineligible, and the remaining 19 were enrolled (Figure 1C). Four participants withdrew consent and discontinued the study during the no-treatment baseline (Stage 1); the remaining 15 began XD-TOMAC (Stage 2) and thus were included in the ITT population. One participant withdrew during Stage 2 due to discomfort associated with the device. The remaining 14 participants completed the study. The final analysis comprised data from the 14 participants who completed the protocol, as well as imputed data for the ITT participant who withdrew.

Table 1 summarizes baseline demographics and clinical characteristics. The mean age of participants was 53.3 years (SD 16.8), and 60% of participants were female. The mean IRLS total score was 29.7 (SD 2.2), with RLS symptoms on 6.9 days per week (SD: 0.5). The mean frequency of midnight RLS symptoms was 5.9 days per week (SD: 1.8), with 67% of participants reporting midnight RLS symptoms 7 nights per week. Of the 15 participants, six were taking medication for RLS, and nine were not (three medication-naïve, six discontinued medications in the past due to inadequate benefit or tolerability). No patients changed RLS medication or dose of medication in the 30 days before entering the study or at any point during the study. Twelve of the fifteen participants met the refractory definition used in the RESTFUL study [14]. The most common concomitant RLS medications were alpha-2-delta ligands (four participants, 27%) and dopamine agonists (two participants, 13%). The mean stimulation intensity was 28.4 mA (SD: 3.6).

### 3.2. Primary and Key Secondary Efficacy Endpoints

**Figure 2:**
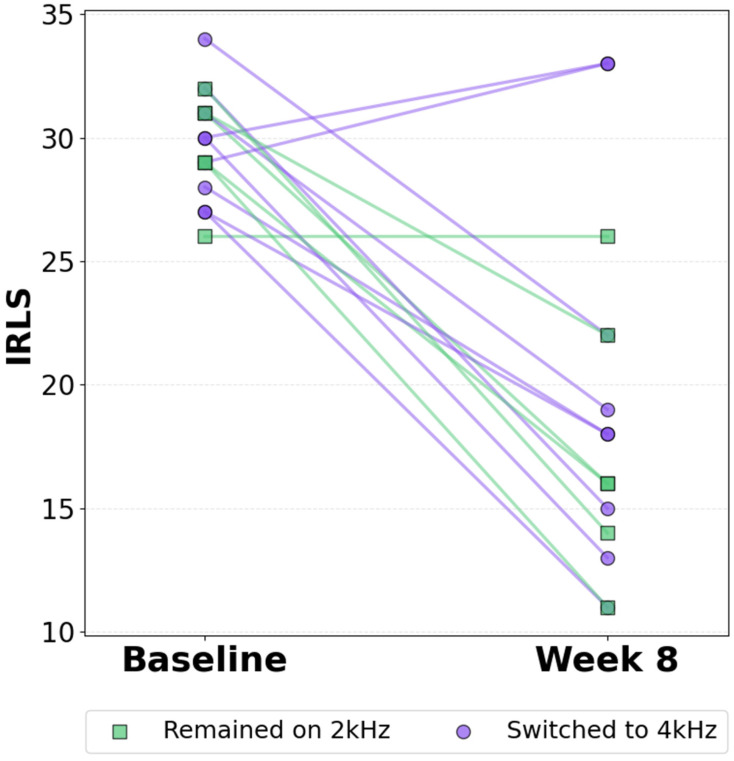
Individual participant change in IRLS scores. Comparison of IRLS score at baseline and week 8 of XD-TOMAC. Each line corresponds to one participant (*n* = 15). Blue corresponds to participants who remained on 2000 Hz XD-TOMAC throughout the study (*n* = 6), and purple corresponds to participants with a Patient Global Impression of Improvement score of ≥3 at week 2 who switched to 4000 Hz for the remainder of the study (*n* = 9).

Primary and key secondary endpoints (Table 2, left column) were assessed at the end of Stage 2 (Week 8 of XD-TOMAC) and compared to study entry (Baseline). The primary endpoint—mean IRLS total score—changed by −10.6 points (CI: −14.3 to −6.9, *p* < 0.001, Cohen’s d = −1.43), demonstrating a reduction in RLS severity with XD-TOMAC. At week 8, the IRLS score was <15 for four patients (27%) and <20 for 10 patients (67%). Figure 2 illustrates individual participant responses. The mean change in frequency of RLS symptoms (days per week) was −0.7 (CI: −1.3 to −0.1, *p* = 0.028, Cohen’s d = −0.57). The mean change in MOS-II total score was −29.5 (CI: −39.4 to 19.7, *p* < 0.001, Cohen’s d = −1.52), and the mean change in MOS-I total score was −27.1 (CI: −36.8 to −17.4, *p* < 0.001, Cohen’s d = −1.41), demonstrating a reduction in sleep problems with XD-TOMAC. The mean PGI-I score at week 8 was 73% (CI: 48% to 89%, *p* < 0.001). These results indicate improvement in RLS and sleep following 8 weeks of XD-TOMAC.

### 3.3. Efficacy Outcomes After the 2-Week Post-Treatment Period

Next, we evaluated the extent to which these improvements persisted at the end of Stage 3 after requiring participants to discontinue XD-TOMAC for 2 weeks (Table 2, right column). Mean IRLS change was −9.3 (CI: −11.9 to −6.6, *p* < 0.001, Cohen’s d = −1.76). Mean change in frequency of RLS symptoms (days per week) was −0.4 (CI: −0.9 to 0.1, *p* = 0.089, Cohen’s d = −0.44). Mean change in MOS-II total score was −24.3 (CI: −34.0 to 14.6, *p* < 0.001, Cohen’s d = −1.27), and mean change in MOS-I total score was −23.3 (CI: −31.0 to −13.0, *p* < 0.001, Cohen’s d = −1.24). PGI-I responder rate was 67% (CI: 42 to 85, *p* < 0.001). These results indicate continued improvement in RLS and sleep following 2 weeks of XD-TOMAC discontinuation.

### 3.4. Sleep Diary Outcomes

**Figure 3:**
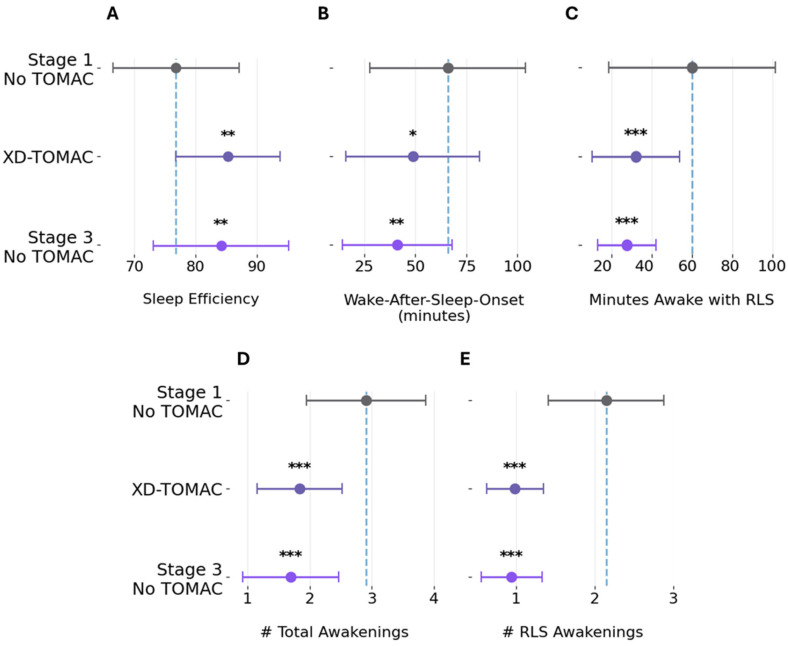
Sleep diary outcomes. (**A**) Sleep efficiency, defined as the percentage of time spent asleep in bed. (**B**) Wake-after-sleep-onset, defined as the number of minutes awake after falling asleep. (**C**) Minutes awake with RLS. (**D**) Number of total awakenings after falling asleep. (**E**) Number of RLS awakenings after falling asleep. Error bars represent standard deviations. * *p* < 0.05. ** *p* < 0.01. *** *p* < 0.001.

Figure 3 summarizes subjective sleep outcomes derived from daily sleep diaries and compared between baseline (Stage 1) and XD-TOMAC (Stage 2). Sleep efficiency improved from 76.8% at baseline to 85.2% during XD-TOMAC (mean difference, MD: +8.5%, 95% CI: +2.3 to +14.6; *p* = 0.001; Cohen’s d = +0.83). Sleep efficiency improvement persisted during the two-week post-treatment period (Stage 3), with a mean increase of +7.4% relative to baseline (95% CI: +0.9 to +13.8; *p* = 0.0081; Cohen’s d = +0.69). Wake after sleep onset (WASO) decreased from 65.9 min at baseline to 48.7 min during XD-TOMAC (MD: −17.2 min; 95% CI: −33.6 to 0.9; *p* = 0.040; Cohen’s d = −0.58). WASO improvement persisted during the post-treatment period (Stage 3), with a mean decrease from baseline of −24.9 min (95% CI: −52.5 to −2.7; *p* = 0.0054; Cohen’s d = −0.50). The number of nocturnal awakenings per night decreased during XD-TOMAC by a mean of −1.1 (95% CI: −1.6 to −0.5; *p* = 0.009; Cohen’s d = −1.1). This improvement persisted during the post-treatment period (mean difference from baseline: −1.2 awakenings; 95% CI: −1.8 to −0.6; *p* = 0.005; Cohen’s d = −1.2). In summary, all subjective sleep outcome measures improved during Stage 2, and these improvements persisted during Stage 3.

Next, we assessed the subset of subjective sleep disruptions that were specifically associated with RLS symptoms according to daily sleep diaries. Time spent awake during the sleep period due to RLS symptoms decreased from 60.0 min at baseline to 32.0 min during XD-TOMAC (mean difference: −28.1 min; 95% CI: −49.6 to −6.6; *p* = 0.009; Cohen’s d = −0.72). This improvement persisted during the two-week post-treatment period (mean difference from baseline: −32.6 min; 95% CI: −65.2 to −0.1; *p* = 0.002; Cohen’s d = −0.55). The mean number of times per night that participants reported being awakened by RLS symptoms also decreased during XD-TOMAC relative to baseline (mean difference: −1.2; 95% CI: −1.7 to −0.6; *p* = 0.003; Cohen’s d = −1.2), with a similar reduction observed during the two-week post-treatment period (mean difference: −1.2; 95% CI: −1.8 to −0.6; *p* = 0.001; Cohen’s d = −1.1). There was no significant change to the mean number of RLS-unrelated awakenings per night during XD-TOMAC relative to baseline (mean difference: +0.1; 95% CI: −0.2 to 0.4; *p* = 0.423; Cohen’s d = 0.21). Item-by-item analysis of the MOS showed consistent findings in Supplementary Table S1. Significant improvements with effect size >0.8 (Cohen’s D) included item 8 (awaken and having trouble falling back asleep), item 1 (time to fall asleep), item 4 (feeling rested upon waking up in the morning), item 6 (feeling drowsy during the day), item 3 (sleep not quiet), and item 12 (getting needed sleep). In summary, participants reported a reduction in subjective sleep disruptions that were associated with RLS symptoms.

### 3.5. Safety and Tolerability

There were no serious or severe device-related adverse events. One participant discontinued treatment due to discomfort associated with wearing the therapy garment during sleep; this discomfort was not related to the electrical stimulation. There were four categories of device-related AEs (Table 3): discomfort (*n* = 4, 27%), skin irritation (*n* = 1, 7%), dry skin (*n* = 1, 7%), and contusion (*n* = 1, 7%). All eight AEs categorized as device-related were rated as mild (Grade 1) by an independent medical monitor. Device-related AEs resolved with minimal intervention; the most common actions taken were adjustment of stimulation intensity and adjustment of therapy unit positioning.

### 3.6. Sensitivity Analysis

Next, we assessed the influence of variable XD-TOMAC output on efficacy. Although all XD-TOMAC sessions were programmed for a runtime duration of 180 min, actual stimulation duration was constrained by the capacity of the rechargeable battery in the TOMAC device, and, thus, actual runtime was affected by the average power required to generate the stimulation waveform. For example, higher titrated stimulation intensity and higher bioimpedance are associated with higher average power output and thus less runtime per battery capacity. Additionally, there was variability in whether participants received the 2000 Hz or 4000 Hz XD-TOMAC stimulation waveform, as discussed in Section 2. Per the study protocol (Section 2), nine participants (60%) were switched from 2000 Hz to 4000 Hz after 2 weeks due to minimal relief from 2000 Hz (PGI-I ≥ 3). Across all participants and all nights, the average stimulation runtime for XD-TOMAC sessions was 159.1 min (SD: 21.8 min), with variability reflecting battery capacity constraints and differences in power consumption required to deliver the stimulation waveform. Stimulation duration was longer on average with 2000 Hz (*n* = 6, mean ± SD: 172.8 ± 6.8 min) than with 4000 Hz (*n* = 9, 155.5 ± 21.8 min, *p* = 0.050), but there was no significant effect of variable runtime on change in IRLS score or MOS-II score either within these two groups or across all participants (*p* > 0.43 for all).

Patients were given the option to manually start additional TOMAC-30 midnight sessions after XD-TOMAC had ended if they awakened with RLS symptoms later in the night. However, only four of the fifteen participants (27%) did this at least once per week, and eight participants (53%) never did this. Supplementary Figure S1 illustrates the initiation times for these sessions, and Supplementary Table S2 shows no significant differences in changes in IRLS or MOS-II scores between participants who used additional midnight sessions and those who did not (*p* > 0.563).

Fourteen out of fifteen participants started XD-TOMAC at bedtime every night, and only one participant had XD-TOMAC initiation delayed by 1.45 h after bedtime to align therapy delivery with their individualized peak window of RLS-related awakenings per protocol (Section 2). The latter patient showed clinically significant improvement in outcome metrics at week 8 of XD-TOMAC, consistent with the group-level results (Supplementary Table S3).

Seven participants reported daytime symptoms more than twice per week at baseline and were instructed to use TOMAC-30 during those periods, consistent with the protocol in RESTFUL. We observed no significant differences in any sleep diary outcomes between this subgroup and participants who reported ≤ 2 days per week of daytime symptoms and did not use TOMAC-30 in addition to XD-TOMAC (*p* > 0.125).

In addition, there was no significant difference primary outcomes (IRLS, MOS-II) between the six participants who were medicated (i.e., taking stable RLS medications in the study) and the nine participants who were non-medicated (i.e., not taking any RLS medications in the study) (medicated IRLS mean −10.5, CI: −17.35 to −3.65; non-medicated IRLS mean −10.67, CI: −16.45 to −4.88, *p* = 0.503; medicated MOS-II mean −27.41, CI: −45.22 to −9.59; non-medicated MOS-II mean −30.93, CI: −46.13 to −15.73, *p* = 0.785; see Supplementary Figure S2).

### 3.7. Comparative Analysis of XD-TOMAC Versus TOMAC-30 Using a Matched Cohort from the RESTFUL Study

Next, we sought to determine if XD-TOMAC led to greater improvements in RLS and sleep compared to TOMAC-30. Since this study did not include a comparator group receiving TOMAC-30 therapy, a matched subset of 45 participants (34%) with frequent RLS symptoms in the RESTFUL study was used as a comparator (see Section 2).

**Figure 4:**
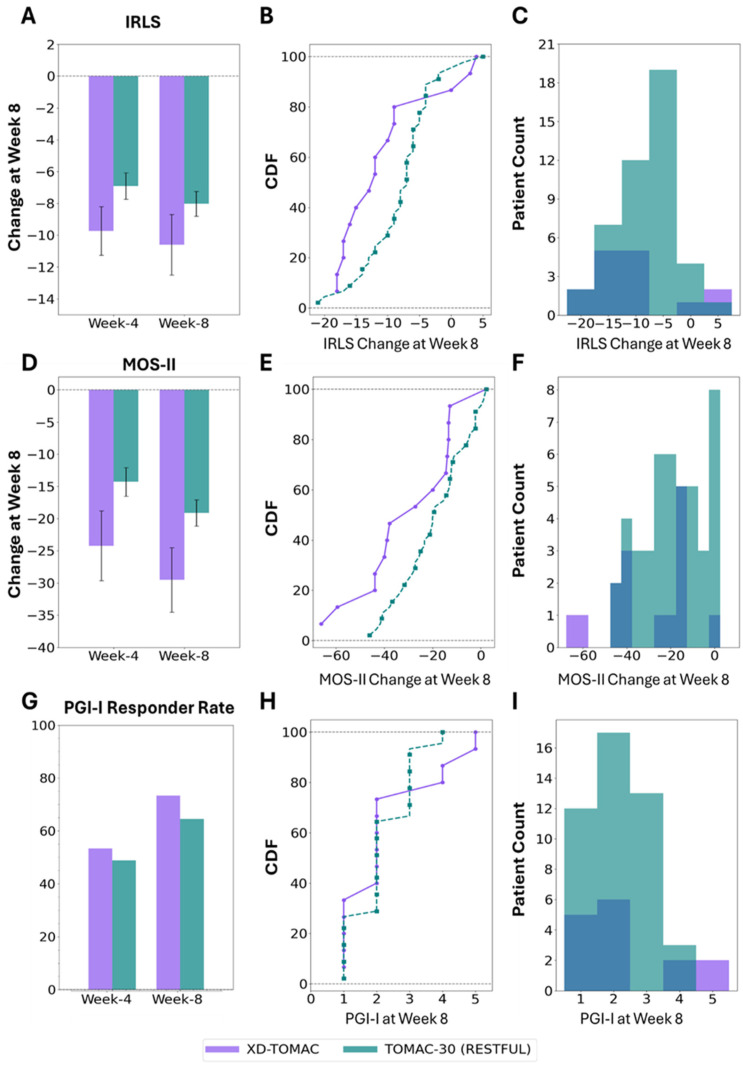
Comparison of XD-TOMAC responses to TOMAC-30 responses in a matched population from the RESTFUL study. (**A**) Change in IRLS total score from study entry. (**B**) CDF and (**C**) histogram plots illustrate the distribution of IRLS change at week 8 of either of the therapy modes. (**D**) Change in MOS-II total score from study entry. (**E**) CDF and (**F**) histogram plots illustrate the distribution of MOS-II change at week 8 of either of the therapy modes. (**G**) PGI-I responder rate at week 8. (**H**) CDF and (**I**) histogram plots illustrate the distribution of PGI-I at week 8 of either of the therapy modes. XD-TOMAC, extended-duration tonic motor activation; IRLS, International Restless Legs Syndrome Study Group Rating Scale; MOS-II, Medical Outcomes Study Sleep Problem Index II; PGI-I, Patient Global Impressions of Improvement; CDF, Cumulative Distribution Function.

Figure 4 summarizes changes in IRLS and MOS-II total scores from study entry to week 8. Detailed results for week 4 and week 8 are provided in Supplementary Table S4. At week 8, mean IRLS improvement was 2.6 points greater for XD-TOMAC compared to TOMAC-30 in RESTFUL (*p* = 0.100). Mean MOS-II improvement was 10.4 points greater for XD-TOMAC than TOMAC-30 (*p* = 0.026). PGI-I responder rate was 9% greater for XD-TOMAC (*p* = 0.527). In summary, XD-TOMAC led to significantly greater improvement in sleep than TOMAC-30 and a trend towards greater improvement in RLS.

While the proportion of any clinically significant improvement (≥3 pts, [18]) for IRLS was similar between XD-TOMAC (80%) and TOMAC-30 (88%), large IRLS improvements (≥10 points) were observed for 67% of XD-TOMAC participants compared to 31% of TOMAC-30 participants (*p* = 0.007), suggesting a shift in the distribution from moderate improvement to large improvement (Kolmogorov–Smirnoff test, *p* = 0.030, Figure 4C). Therefore, XD-TOMAC was more likely to result in large improvements in RLS than TOMAC-AN.

## 4. Discussion

These results indicate that XD-TOMAC is efficacious and often leads to large reductions in RLS severity and sleep problems. Benchmarked against a matched cohort from the RESTFUL study, XD-TOMAC was associated with a much higher likelihood of ≥10-point reductions in IRLS and ≥30-point improvements in MOS sleep. There was a consistent effect of XD-TOMAC on reducing midnight awakenings, as demonstrated by improvements in sleep diary outcomes (reduced WASO and number of awakenings) and MOS sleep item 8 (disruptive awakenings). The suppression of midnight awakenings was specific to RLS-related awakenings; XD-TOMAC reduced the overall number of awakenings (delta: −1.1) and RLS-related number of awakenings (delta: −1.2) but did not change RLS-unrelated number of awakenings (+0.1). XD-TOMAC resulted in significant improvements in multiple other dimensions of sleep that have also been observed with TOMAC-30, including reductions in daytime sleepiness and sleep onset latency. The observed reduction in time spent awake due to RLS symptoms (28.1 min) and overall reduction in WASO (17.1 min) are both larger than the minimal clinically important difference for WASO (10 min) [19]. These findings suggest that delivering TOMAC stimulation for a longer duration during the sleep period may be particularly advantageous for patients whose RLS predominantly disrupts sleep continuity. The 180 min duration studied here was limited by battery capacity, and it remains possible that even longer durations could lead to greater benefits.

XD-TOMAC was well-tolerated and safe, with a risk profile equivalent to prior studies of TOMAC-30 [14]. Whereas prior studies demonstrated that patients could fall asleep during 30 min of TOMAC stimulation [16], the present study is the first to show that patients can remain asleep throughout a longer duration of TOMAC stimulation. The average duration of XD-TOMAC (159 min) was longer than the typical duration of the first sleep cycle (approximately 90 min [20]), suggesting that TOMAC may be compatible with each sleep stage. No participants discontinued due to adverse effects related to electrical stimulation, no participants reported sleep disruption or worsening during XD-TOMAC stimulation, and there was no evidence of an increased frequency or severity of device-related adverse events compared to prior investigations of TOMAC-30.

Therapeutic effects were sustained beyond the active treatment period, with statistically significant improvements in RLS severity and sleep outcomes persisting for two weeks following cessation of XD-TOMAC. Furthermore, there was no evidence of reversion in any of the improvements after the two-week post-treatment period. Prior reports have shown that 2 weeks of TOMAC-30 results in no carryover after 2 weeks post-treatment [13] and 32 weeks of TOMAC-30 results in partial carryover after 8 weeks post-treatment [15]. Further studies would be helpful to assess and compare the duration of the carryover effect for 8 weeks of XD-TOMAC and/or TOMAC-30. One possibility that remains to be explored is that long-term exposure to TOMAC leads to dose-dependent neural plasticity that mediates these carryover effects. This could explain why longer stimulation sessions (180 vs. 30 min) and longer duration of usage (32 weeks vs. 2 weeks) would lead to greater carryover effects. If this explanation is correct, XD-TOMAC might be expected to confer benefits over TOMAC-30, even for patients who do not have frequent midnight awakenings.

There are a number of limitations to this study. An internal TOMAC-30 comparator group would be a better way to compare XD-TOMAC with TOMAC-30. An internal sham control group would have been helpful to determine the contribution of the placebo effect and Hawthorne effect [21], although this can be estimated based on prior randomized controlled trials of TOMAC-30. An important distinction between XD-TOMAC and the TOMAC-30 approach is the timing of therapy delivery (at bedtime every night vs. as needed at symptom onset). It remains unclear whether extended-duration stimulation applied as needed at symptom onset would produce similar effects, and future studies would be helpful to disentangle the relative contributions of timing versus duration. A longer duration post-treatment period would be helpful to explore the durability of the sustained benefits after XD-TOMAC cessation. There was heterogeneity in the patient population (e.g., medication-refractory vs. naïve), run-in period, and stimulation frequency. Although this study evaluates an investigational device (not a commercial product), the study was conducted by employees of the TOMAC device manufacturer, and, thus, potential bias should be considered. There are no validated measures to quantify WASO related to RLS, and, thus, custom sleep diaries were utilized for some of the exploratory endpoints; the validity and repeatability of these measures have not been established. The bimodal distribution of RLS improvement with XD-TOMAC raises interesting possibilities; it would be especially helpful to identify clinical factors predicting which patients will be strong responders to XD-TOMAC. Future studies should address these limitations.

## 5. Conclusions

In conclusion, bilateral high-frequency peroneal nerve stimulation applied for a duration of approximately 3 h starting at bedtime is a promising therapeutic option for RLS patients whose symptoms include frequent midnight awakenings. Further studies would be helpful to evaluate XD-TOMAC in RLS patients without frequent midnight awakenings and in special populations such as pregnant women, adolescents, and children.

## Figures and Tables

**Table 1:** Demographic and clinical characteristics.

	ITT (*n* = 15)
Age, years, mean (SD)	53.3 (16.8)
Sex, *n* (%)	
Male	6 (40)
Female	9 (60)
Race/ethnicity, *n* (%)	
White, non-Hispanic	12 (80)
White, Hispanic	1 (6)
Black or African American	1 (6)
Asian	1 (6)
IRLS score, mean (SD)	29.7 (2.2)
Years with RLS, years, mean (SD)	11.5 (9.9)
MOS, mean (SD)	
MOS-I Sleep Problems Index	79.3 (12.8)
MOS-II Sleep Problems Index	67.1 (13.2)
Frequency of Symptoms per Week, mean (SD)	
RLS symptoms	6.9 (0.5)
Midnight RLS symptoms	5.9 (1.8)
RLS medications, *n* (%)	
None	9 (60)
Alpha-2-delta ligands	4 (27)
Dopamine agonists	2 (13)
Benzodiazepines	1 (7)
Opioid	1 (7)
Stimulation intensity (mA), mean (SD)	28.4 (3.6)

Abbreviations. ITT, modified intention to treat; SD, standard deviation; IRLS, International RLS Study Group Rating Scale; RLS, restless legs syndrome.

**Table 2:** Primary and key secondary efficacy endpoints.

	Primary Analysis at End of Stage 2 (Week 8 of XD-TOMAC) vs. Baseline	Exploratory Analysis at End of Stage 3 (After 2 Weeks of XD-TOMAC Discontinuation) vs. Baseline
**Primary Endpoint**		
Change in IRLS score		
*n*	15	15
Mean	−10.6	−9.3
SD	7.4	5.3
Min, Max	−18, 4	−16, 2
95% CI	−14.3 to −6.9	−11.9 to −6.6
*p*-value	<0.001	<0.001
Effect size	−1.43	−1.76
**Key Secondary Endpoints**		
Frequency of RLS symptoms (days per week)		
*n*	15	15
Mean	−0.7	−0.4
SD	1.2	0.9
Min, Max	−4, 0	−3, 0
95% CI	−1.3 to −0.1	−0.9 to 0.1
*p*-value	0.028	0.089
Cohen’s d	−0.57	−0.44
Change in MOS-II score		
*n*	15	15
Mean	−29.5	−24.3
SD	19.4	19.2
Min, Max	−66.1, 2.2	−61.7, 1.7
95% CI	−39.4 to 19.7	−34.0 to −14.6
*p*-value	<0.001	<0.001
Cohen’s d	−1.52	−1.27
Change in MOS-I score		
*n*	15	15
Mean	−27.1	−22.0
SD	19.2	17.8
Min, Max	−60.0, 3.3	−60.0, 6.7
95% CI	−36.8 to −17.4	−31.0 to −13.0
*p*-value	<0.001	<0.001
Cohen’s d	−1.41	−1.24
PGI-I Responder		
*n*	15	15
Responder %	73	67
Responder *n*	11	10
95% CI	48 to 89	42 to 85
*p*-value	<0.001	<0.001

Abbreviations: IRLS, International Restless Legs Syndrome Study Group Rating Scale; MOS-I, Medical Outcomes Study Sleep Problem Index I; MOS-II, Medical Outcomes Study Sleep Problem Index II; PGI-I, Patient Global Impressions of Improvement; SD, standard deviation.

**Table 3:** Treatment-emergent adverse events.

	Number (%) of Participants	
	All AEs	Device-Related AEs
Any AE	9 (60.0)	7 (46.7)
Serious AE	0	0
AE severity		
Grade 1	9 (60.0)	7 (46.7)
Grade 2 or higher	0	0
MedDRA preferred term		
Discomfort	4 (26.6)	4 (26.6)
Skin irritation	1 (6.6)	1 (6.6)
Dry skin	1 (6.6)	1 (6.6)
Contusion	1 (6.6)	1 (6.6)
Abdominal discomfort	1 (6.6)	0
Back pain	1 (6.6)	0
Vomiting	1 (6.6)	0

Abbreviations: AE, adverse event; MedDRA, Medical Dictionary for Regulatory Activities.

## Data Availability

The deidentified data that support the findings of this study are available from the corresponding author upon reasonable request.

## Supplementary Materials

The following supporting information can be downloaded at: https://www.mdpi.com/article/10.3390/jcm15082845/s1, Section S1: Subjective Sleep Assessment Questions; Table S1: MOS sleep item-by-item responses; Table S2: Sensitivity analysis for the use of additional midnight TOMAC-30 sessions after XD-TOMAC; Table S3: Primary and key secondary outcome metrics for the patient with delayed XD-TOMAC; Table S4: Primary and key secondary outcome metrics at weeks 4 and 8 for XD-TOMAC and TOMAC-30 (RESTFUL); Figure S1: Distribution of TOMAC session start times over the night; Figure S2: Comparison of medicated versus non-medicated subgroups at week -8 of XD-TOMAC.

## Abbreviations

The following abbreviations are used in this manuscript:

RLS	Restless Legs Syndrome
TOMAC	Tonic Motor Activation
XD-TOMAC	Extended-Duration Tonic Motor Activation
IRLS	International RLS Study Group Rating Scale
MOS	Medical Outcomes Study
AASM	American Academy of Sleep Medicine
PGI-I	Participant Global Impressions of Improvement
SOL	Sleep Onset Latency
WASO	Wake After Sleep Onset
TIB	Time in Bed
TST	Total Sleep Time
SE	Sleep Efficiency
ITT	Intention To Treat
ANCOVA	Analysis of Covariance
SD	Standard Deviation
CI	Confidence Interval
AE	Adverse Event
NINDS	National Institute of Neurological Disorders and Stroke

## References

[1] Abetz L., Allen R., Follet A., Washburn T., Early C., Kirsch J., Knight H. (2004). Evaluating the quality of life of patients with restless legs syndrome. Clin. Ther..

[2] Bogan R.K. (2006). Effects of restless legs syndrome (RLS) on sleep. Neuropsychiatr. Dis. Treat..

[3] Zucconi M., Manconi M. (2008). Sleep and Quality of Life in Restless Legs Syndrome.

[4] Zhang H., Zhang Y., Ren R., Yang L., Shi Y., Vitiello M.V., Sanford L.D., Tang X. (2022). Polysomnographic features of idiopathic restless legs syndrome: A systematic review and meta-analysis of 13 sleep parameters and 23 leg movement parameters. J. Clin. Sleep Med..

[5] Mullington J.M., Haack M., Toth M., Serrador J.M., Meier-Ewert H.K. (2009). Cardiovascular, inflammatory, and metabolic consequences of sleep deprivation. Prog. Cardiovasc. Dis..

[6] Reynolds A.C., Banks S. (2010). Total sleep deprivation, chronic sleep restriction and sleep disruption. Prog. Brain Res..

[7] Silber M.H., Buchfuhrer M.J., Earley C.J., Koo B.B., Manconi M., Winkelman J.W. (2021). Scientific and Medical Advisory Board of the Restless Legs Syndrome Foundation. The management of restless legs syndrome: An updated algorithm. Mayo Clin. Proc..

[8] Winkelman J.W., Berkowski J.A., DelRosso L.M., Koo B.B., Scharf M.T., Sharon D., Zak R.S., Kazmi U., Falck-Ytter Y., Shelgikar A.V. (2025). Treatment of restless legs syndrome and periodic limb movement disorder: An American Academy of Sleep Medicine clinical practice guideline. J. Clin. Sleep Med..

[9] U.S. Food and Drug Administration (2019). FDA Warns About Serious Breathing Problems with Gabapentin and Pregabalin.

[10] Oertel W.H., Hallström Y., Saletu-Zyhlarz G.M., Hopp M., Bosse B., Trenkwalder C., RELOXYN Study Group (2016). Sleep and quality of life under prolonged-release oxycodone–naloxone for severe restless legs syndrome: An analysis of secondary efficacy variables of a double-blind, randomized, placebo-controlled study with an open-label extension. CNS Drugs.

[11] Trenkwalder C., Beneš H., Grote L., García-Borreguero D., Högl B., Hopp M., Bosse B., Oksche A., Reimer K., RELOXYN Study Group (2013). Prolonged-release oxycodone–naloxone for treatment of severe restless legs syndrome after failure of previous treatment: A double-blind, randomised, placebo-controlled trial with an open-label extension. Lancet Neurol..

[12] Charlesworth J.D., Adlou B., Singh H., Buchfuhrer M.J. (2023). Bilateral high-frequency noninvasive peroneal nerve stimulation evokes tonic leg muscle activation for sleep-compatible reduction of restless legs syndrome symptoms. J. Clin. Sleep Med..

[13] Buchfuhrer M.J., Baker F.C., Singh H., Kolotovska V., Adlou B., Anand H., de Zambotti M., Ismail M., Raghunathan S. (2021). Noninvasive neuromodulation reduces symptoms of restless legs syndrome. J. Clin. Sleep Med..

[14] Bogan R.K., Roy A., Kram J., Ojile J., Rosenberg R., Hudson J.D., Scheuller H.S., Winkelman J.W., Charlesworth J.D. (2023). Efficacy and safety of tonic motor activation (TOMAC) for medication-refractory restless legs syndrome: A randomized clinical trial. Sleep.

[15] Roy A., Ojile J., Kram J., Olin J., Rosenberg R., Hudson J.D., Bogan R.K., Charlesworth J.D. (2023). Long-term efficacy and safety of tonic motor activation for treatment of medication-refractory restless legs syndrome: A 24-week open-label extension study. Sleep.

[16] Rigot S., Adlou B., Kolotovska V., Tekchandani J., Charlesworth J.D. (2024). Tonic motor activation for restless legs syndrome is compatible with sleep onset and reduces periodic leg movements. Sleep.

[17] Allen R.P., Picchietti D.L., Garcia-Borreguero D., Ondo W.G., Walters A.S., Winkelman J.W., Zucconi M., Ferri R., Trenkwalder C., International Restless Legs Syndrome Study Group (2014). Restless legs syndrome/Willis–Ekbom disease diagnostic criteria: Updated International Restless Legs Syndrome Study Group (IRLSSG) consensus criteria—History, rationale, description, and significance. Sleep Med..

[18] Allen R.P. (2013). Minimal clinically significant change for the International Restless Legs Syndrome Study Group rating scale in clinical trials is a score of 3. Sleep Med..

[19] Winkelman J.W., Berkowski J.A., DelRosso L.M., Koo B.B., Scharf M.T., Sharon D., Zak R.S., Kazmi U., Carandang G., Falck-Ytter Y. (2025). Treatment of restless legs syndrome and periodic limb movement disorder: An American Academy of Sleep Medicine systematic review, meta-analysis, and GRADE assessment. J. Clin. Sleep Med. JCSM Off. Publ. Am. Acad. Sleep Med..

[20] Patel A.K., Reddy V., Shumway K.R., Araujo J.F. (2024). Physiology, sleep stages. StatPearls.

[21] Wickström G., Bendix T. (2000). The “Hawthorne effect”—What did the original Hawthorne studies actually show?. Scand. J. Work Environ. Health.

