# Supplementary material for: Efficacy and Tolerability of Extended-Duration Tonic Motor Activation for Treatment of Restless Legs Syndrome with Awakenings During Sleep
Source: J Clin Med. 2026 Apr 9;15(8):2845. doi: 10.3390/jcm15082845 (PMC13116870; doi:10.3390/jcm15082845)
Supplement: Supplementary file 1 [file jcm-15-02845-s001.zip › jcm-4179987-supplementary.pdf]

Supplementary Material Section S1: Subjective Sleep Assessment Questions

- What time did you try to go to sleep last night ("bedtime")?
- What time was your final awakening ("morning wake up time")?
- From your bedtime, how many minutes did it take you to fall asleep? (specify one whole number, to your best recollection)
- How many times did you wake up after you fell asleep and before you woke up for the next day in the morning? − <u>regardless whether you had RLS symptoms or not </u>(specify one whole number, to your best recollection)
- How many total minutes were you awake in the middle of the night after you first fell asleep and before your final awakening in the morning − <u>regardless whether you had RLS or not</u>? (specify a number in increments of 15 minutes)
- How many times did you wake up − **<u>With RLS Symptoms </u>**− after you fell asleep and before you woke up for the next day in the morning? (specify one whole number, to your best recollection)
- How many total minutes were you awake **− <u>with RLS Symptoms</u> −** in the middle of the night after you first fell asleep and before your final awakening in the morning? (specify a whole number in increments of 15 minutes)
- IRLS Question 7 Follow−up: For example, for a response of 2) Moderate 2−3 days per week, the subject will be asked to select between 2 or 3 days per week." Sample response: 2 days

Supplementary Table S1: MOS sleep item−by−item responses.

Item #	Category	Mean at Study Entry	Mean Change at Week 8 of XD−TOMAC	Cohen's D	*p*−Value
8	Awaken and having trouble falling back asleep	72.0	−37.3	−1.32	<0.001
1	Time to fall asleep	71.7	−41.7	−1.15	<0.001
4	Feeling rested	13.3	+32.0	−1.14	<0.001
6	Feeling drowsy during day	81.3	−34.7	−0.99	<0.001
3	Sleep not quiet	74.7	−26.7	−0.86	<0.001

12	Getting needed sleep	13.3	+30.7	+0.85	0.001
7	Trouble falling asleep	70.7	−28.0	−0.81	0.002
5	Shortness of breath	22.7	−21.3	−0.80	0.002
9	Trouble staying awake during the day	37.3	−13.3	−0.42	0.104
10	Snoring	38.7	−9.3	−0.41	0.108
2	Number of hours of sleep	5.6 hr	+0.7 hr	+0.36	0.090
11	Taking naps during day	21.3	+5.3	+0.26	0.317

Supplementary Table S2: Sensitivity analysis for the use of additional midnight TOMAC−30 sessions after XD−TOMAC

	**<1 midnight session/week**	**With >=1 midnight session/week**
**Change in IRLS Score**		
n	11	4
Mean	−10.36	−11.25
SD	6.70	10.24
95% CI	−14.86 to −5.86	−27.55 to 5.05
p−value		0.879
Cohen's d		0.12
**Change in MOS−II Score**		
n	11	4
Mean	−31.57	−23.89
SD	19.18	21.92
95% CI	−44.45 to −18.68	−58.77 to 10.99
p−value		0.563
Cohen's d		−0.39

Supplementary Table S3: Primary and key secondary outcome metrics for the patient with delayed XD–TOMAC

	Baseline at Study Entry	Week 8 of XD–TOMAC vs Baseline
**Primary Endpoint**		
Change in IRLS total score	32.0	−17.0
**Key Secondary Endpoints**		
Change in frequency of RLS symptoms (days/week)	7.0	0
Change in MOS–II total score	93.3	−66.1
Change in MOS–I total score	81.7	−60.0
PGI–I Responder	na	Much Improved

Supplementary Table S4: Primary and key secondary outcome metrics at weeks 4 and 8 for XD–TOMAC and TOMAC–30 (RESTFUL)

	Week 4 versus Baseline		Week 8 versus Baseline	
	**XD–TOMAC**	**TOMAC–30 (RESTFUL)**	**XD–TOMAC**	**TOMAC–30 (RESTFUL)**
**Primary Endpoint**				
Change in IRLS total score				
n	15	45	15	45
Mean	−9.7	−6.9	−10.6	−8.0
SD	5.9	5.6	7.4	5.3
Min, Max	−20, 0	−23.0. 3.0	−18, −12	−21.0, 5.0
95% CI	−13.0 to −6.6	−8.5 to −5.3	−14.3 to −6.9	−9.6 to −6.5
p−value	<0.001	<0.001	<0.001	<0.001
Cohen's d	−1.65	−1.24	−1.43	−1.5
**Key Secondary Endpoints**				
Change in MOS–II total score				

n	15	45	15	45
Mean	−24.2	−14.3	−29.5	−19.1
SD	21.1	14.9	19.4	13.8
Min, Max	−63.9, 5.0	−61.1 to 10.0	−66.1, 2.2	−46.1, 2.2
95% CI	−35.0 to −14.6	−18.6 to −9.9	−39.4 to 19.7	−23.1 to −15.1
p−value	<0.001	<0.001	<0.001	<0.001
Cohen's d	−1.15	−0.96	−1.52	−1.39
Change in MOS−I total score				
n	15	45	15	45
Mean	−24.2	−12.9	−27.1	−16.6
SD	20.3	15.2	19.2	14.3
Min, Max	−63.3, 3.3	−60.0, 13.3	−60.0, 3.3	−43.3, 13.3
95% CI	−34.5 to −14	−17.3 to −8.4	−36.8 to −17.4	−20.8 to −12.4
p−value	<0.001	<0.001	<0.001	<0.001
Cohen's d	−1.2	−0.84	−1.41	1.16
PGI−I Responder				
n	15	45	15	45
Responder %	53.3	48.8	73.3	64.4
Responder n	8	22	11	29
95% CI	30 to 75	34.9 to 63.0	48.0 to 89.1	49.8 to 76.8
p−value	<0.001	<0.001	<0.001	<0.001

Supplementary Figure S1: Distribution of TOMAC session start times over the night.

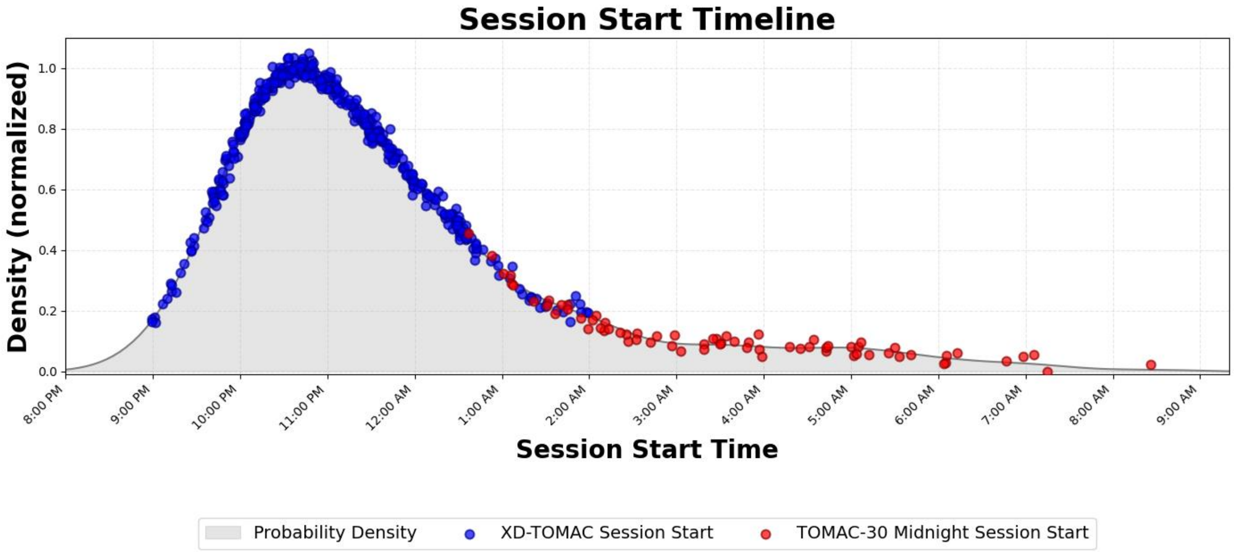

Supplementary Figure S2: Comparison of medicated versus non–medicated subgroups at week 8 of XD–TOMAC

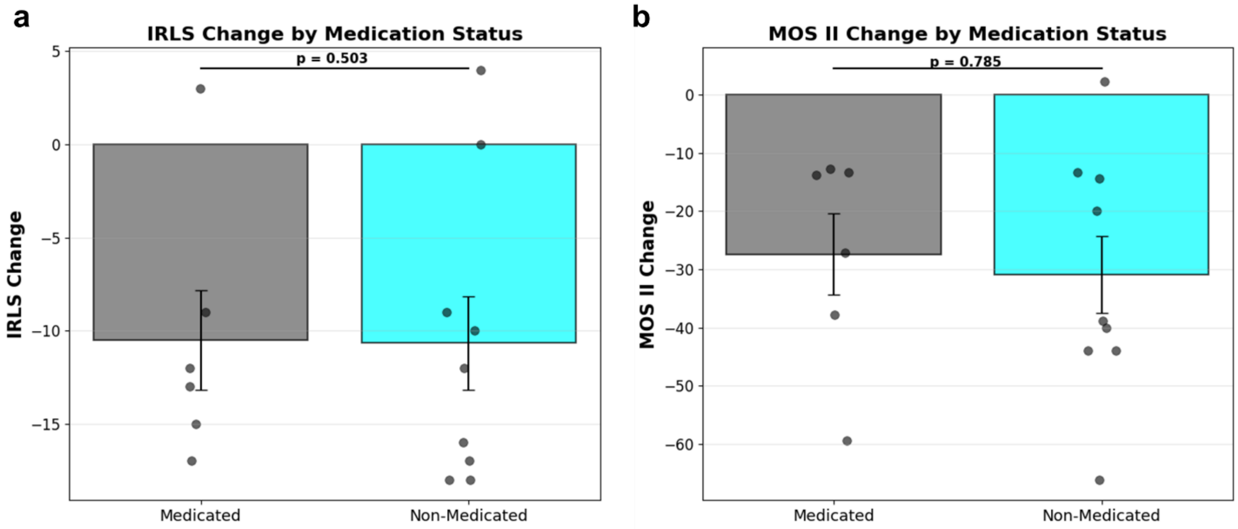

Figure S2. Subgroup analysis for medicated (n=6) versus non–medicated (n=9) participants at week 8 of XD–TOMAC. (a) Change in IRLS score. (b) Change in MOS–II score.
